# Genetic variation in the *lactase* gene, dairy product intake and risk for prostate cancer in the European Prospective Investigation into Cancer and Nutrition

**DOI:** 10.1002/ijc.27836

**Published:** 2012-09-11

**Authors:** Ruth C Travis, Paul N Appleby, Afshan Siddiq, Naomi E Allen, Rudolf Kaaks, Federico Canzian, Silke Feller, Anne Tjønneland, Nina Føns Johnsen, Kim Overvad, J Ramón Quirós, Carlos A González, Maria-José Sánchez, Nerea Larrañaga, Maria-Dolores Chirlaque, Aurelio Barricarte, Kay-Tee Khaw, Nick Wareham, Antonia Trichopoulou, Elisavet Valanou, Erifili Oustoglou, Domenico Palli, Sabina Sieri, Rosario Tumino, Carlotta Sacerdote, H B(as) Bueno-de-Mesquita, Pär Stattin, Pietro Ferrari, Mattias Johansson, Teresa Norat, Elio Riboli, Timothy J Key

**Affiliations:** 1 Cancer Epidemiology Unit, Nuffield Department of Clinical Medicine, University of OxfordOxford, United Kingdom; 2 Department of Epidemiology and Public Health, Imperial CollegeLondon, United Kingdom; 3 Division of Cancer Epidemiology, German Cancer Research CentreHeidelberg, Germany; 4 Department of Epidemiology, German Institute of Human NutritionPotsdam-Rehbruecke, Nuthetal, Germany; 5 Institute of Cancer Epidemiology, Danish Cancer SocietyCopenhagen, Denmark; 6 Department of Cardiology, Center for Cardiovascular Research, Aalborg Hospital, Aarhus University Hospital and Department of Epidemiology, School of Public Health, Aarhus UniversityAarhus, Denmark; 7 ^7^Public Health DirectorateAsturias, Spain; 8 Unit of Nutrition, Environment and Cancer, Cancer Epidemiology Research Programme, Catalan Institute of OncologyBarcelona, Spain; 9 Andalusian School of Public Health, Granada (Spain) and CIBER de Epidemiología y Salud Pública (CIBERESP)Spain; 10 Public Health Division of Gipuzkoa, Basque Regional Health Department and Ciberesp-BiodonostiaSan Sebastian, Spain; 11 Epidemiology Department, Murcia Health Council, CIBER en Epidemiología y Salud, Pública (CIBERESP)Murcia, Spain; 12 Navarre Public Health Institute, Pamplona, Spain and Consortium for Biomedical Research in Epidemiology and Public Health (CIBER Epidemiología y Salud Pública-CIBERESP)Spain; 13 University of CambridgeUnited Kingdom; 14 MRC Epidemiology UnitCambridge, United Kingdom; 15 WHO Collaborating Center for Food and Nutrition Policies, Department of Hygiene, Epidemiology and Medical Statistics, University of Athens Medical SchoolAthens, Greece; 16 Hellenic Health FoundationAthens, Greece; 17 Molecular and Nutritional Epidemiology Unit, Cancer Research and Prevention Institute, ISPOFlorence Italy; 18 Nutritional Epidemiology Unit, Fondazione IRCCS Istituto Nazionale TumoriMilan, Italy; 19 Cancer Registry and Histopathology Unit, “Civile - M.P.Arezzo” HospitalASP 7 Ragusa, Italy; 20 Center for Cancer Prevention (CPO-Piemonte), and Human Genetic Foundation (HuGeF)Torino, Italy; 21 National Institute for Public Health and the Environment (RIVM)Bilthoven, The Netherlands; 22 Department of Gastroenterology and Hepatology, University Medical CentreUtrecht, The Netherlands; 23 Department of Public Health and Clinical Medicine, Nutritional Research, Umeå University, Umeå, Sweden/ Department of Surgical and Perioperative Sciences, Urology and Andrology, Umeå UniversityUmeå, Sweden; 24 Nutrition and Metabolism Section, International Agency for Research on Cancer, The World Health OrganizationLyon, France; 25 International Agency for Research on Cancer (IARC)Lyon, France; 26 Department of Surgical and Perioperative Sciences, Urology and Andrology, Umeå UniversityUmeå, Sweden

**Keywords:** prostate cancer, polymorphism, genetic, lactase, dairy

## Abstract

High dairy protein intake has been found to be associated with increased prostate cancer risk in the European Prospective Investigation into Cancer and Nutrition (EPIC). To further examine this possible relationship, we investigated the hypothesis that a genetic polymorphism in the *lactase* (*LCT*) gene might be associated with elevated dairy product intake and increased prostate cancer risk in a case–control study nested in EPIC. The C/T-13910 *lactase* variant (rs4988235) was genotyped in 630 men with prostate cancer and 873 matched control participants. Dairy product consumption was assessed by diet questionnaire. Odds ratios (ORs) for prostate cancer in relation to *lactase* genotype were estimated by conditional logistic regression. *Lactase* genotype frequency varied significantly between countries, with frequencies of the T (lactase persistence) allele ranging from 7% in Greece to 79% in Denmark. Intake of milk and total dairy products varied significantly by *lactase* genotype after adjustment for recruitment center; adjusted mean intakes of milk were 44.4, 69.8 and 82.3 g/day among men with CC, CT and TT genotypes, respectively. The *lactase* variant was not significantly associated with prostate cancer risk, both in our data (adjusted OR for TT vs. CC homozygotes: 1.10, 95% CI: 0.76–1.59) and in a meta-analysis of all the published data (combined OR for T allele carriers vs. CC homozygotes: 1.12, 0.96–1.32). These findings show that while variation in the *lactase* gene is associated with milk intake in men, the *lactase* polymorphism does not have a large effect on prostate cancer risk.

**What's new?**

High dairy protein intake has previously been found to be associated with increased prostate cancer risk in the European Prospective Investigation into Cancer and Nutrition (EPIC). The current study was nested in EPIC, and results from this first Europe-wide study suggest that while the C/T13910 lactase polymorphism is associated with milk intake, the variant has no large effect on prostate cancer risk. The data illustrate the challenges of applying mendelian randomisation to explore the relationship between dairy product consumption and cancer risk. Very large studies with both genetic and dietary data are thus needed for investigations using genetic proxies of nutritional exposures.

A high intake of dairy protein has been found to be associated with increased risk for prostate cancer in the European Prospective Investigation into Cancer and Nutrition,^1^ although results from other prospective studies of dairy products have been inconsistent.^2^–^9^ The inconsistencies in the published literature may be in part due to measurement error, with estimates of dairy product intake based on self-reported information obtained by dietary questionnaire. It is also possible that the associations found with dairy products and prostate cancer risks are due to confounding by unknown dietary or lifestyle factors.

The main sugar in dairy products is lactose, and the digestion and absorption of lactose is dependent on the activity of the lactase enzyme (lactase-phlorizin hydrolase) in the wall of the intestine. A C/T polymorphism (rs4988235), which resides 13,910 base pairs upstream of the lactase coding sequence in the *lactase* gene, is the major variant associated with activity of the lactase enzyme in European populations.^10^ The ancestral C allele is associated with the developmental downregulation of the lactase enzyme after the first few years of life (also referred to as lactase nonpersistence) and with reduced tolerance of lactose-rich foods in adulthood.^11^ The T variant is associated with continued lactase production in adulthood (lactase-persistance) and is thought to have arisen around 9000 years ago and become common in northern Europe, probably due to the selective advantage conferred by dairy consumption in the context of the spread of dairy farming.^12^ To date, however, the published epidemiological data on dairy product consumption in adulthood by *lactase* genotype is limited.^13^–^15^

The existence of the *lactase* C/T polymorphism and the limited evidence suggesting a link with dairy product consumption provide an opportunity to explore the Mendelian randomization approach, using *lactase* genotype as a proxy for dairy intake and exploiting the random allocation of alleles from parents to offspring, to overcome potential confounding of the association between dairy product consumption and cancer risk.^16^–^18^ The proposed hypothesis is that individuals carrying the T allele (lactase-persistence variant) will have a higher intake of lactose-rich dairy products and an increased risk for prostate cancer.

We report here findings from a Europe-wide study of the C/T-13910 *lactase* genotypes in relation to intake of dairy products and risk for prostate cancer, among 630 men with incident prostate cancer and 873 matched control participants participating in the European Prospective Investigation into Cancer and Nutrition (EPIC).

## Material and Methods

### Participants and data

Between 1992 and 2000, approximately 500,000 individuals (150,000 men) were recruited into the European Prospective Investigation into Cancer and Nutrition (EPIC) from 23 centers in 10 European countries. The methods of recruitment and study design have been described in detail elsewhere.^19^ Participants completed an extensive questionnaire on dietary and nondietary factors at recruitment, and about 400,000 individuals (of whom 137,000 were men) also provided a blood sample. All participants gave written consent, and approval for the study was obtained from the Internal Review Board of the International Agency for Research on Cancer (Lyon, France) and from the local ethics committees in participating countries.

Dietary intake during the year before enrolment was measured by country-specific validated food frequency questionnaires or diet histories, as previously described.^19^ For this analysis, dairy products included milk and milk beverages, yoghurt, fromage blanc, cheese, dairy puddings/desserts, dairy creams and butter. Intakes were calculated as g/day. Estimated daily nutrient intakes were calculated by multiplying the nutrient content of each food of a specific portion size by the frequency of consumption as stated on the dietary questionnaire using national food tables from each country as compiled in the EPIC Nutrient DataBase' (ENDB).^20^,^21^ Measurements of circulating concentrations of certain nutritional and hormonal analytes have been made in previous nested case control studies of prostate cancer in men from EPIC and were available for the current analyses.^22^–^26^

This study includes prostate cancer cases occurring after blood collection and individually matched male control participants from the eight participating countries which recruited men: Denmark, Germany, Greece, Italy, the Netherlands, Spain, Sweden and the United Kingdom (UK).

Follow-up for diagnosis of prostate cancer is provided through record linkage with population-based cancer registries in six of the participating countries: Denmark, Italy, the Netherlands, Spain, Sweden and the UK. In Germany and Greece, follow-up is active and is achieved through checks of insurance records and cancer and pathology registries as well as *via* self-reported questionnaires; self-reported incident cancers are verified through medical records. Data on vital status in most EPIC study centers were collected from mortality registries at the regional or national level, in combination with data collected by active follow-up (Greece). The 10th Revision of the International Statistical Classification of Diseases, Injuries and Causes of Death (ICD) was used, and cancer of the prostate was defined as code C61. For each EPIC center closure dates of the study period were defined as the latest dates of complete follow-up for both cancer incidence and vital status (dates varied between centers, from June 1999 to January 2003).

Case patients were men who developed prostate cancer after the date of blood collection and before the end of the study period, defined for each study center by the latest date of follow-up. The cases with no available blood sample and those participants who had missing information on the date of the blood donation or who had a history of another cancer (except nonmelanoma skin cancer) at the time of the blood collection were excluded. After these exclusions, at the time of genotyping for the current study, DNA was available for approximately 600 men with prostate cancer who had had their DNA extracted for the EPIC component of the Breast and Prostate Cancer Cohort Consortium.

Data on the stage and grade of disease at diagnosis were collected from each center, where possible. A total of 480 cases (76.7%) had information on tumor stage; of these 328 (52.6%) were classified as localized (tumor [T], node [N], metastasis [M] categories T0 or T1 or T2 and N0 or NX and M0, or stage coded in recruitment center as localized), and 152 (24.1%) were classified as advanced (T3 or T4, N1+, M1, or some combination of these, or stage coded in recruitment center as metastatic). Information on histological grade was available for 423 cases (65.2%); of these, 301 (47.8%) were classified as low-grade (Gleason sum <7 or equivalent, *i.e*. coded as moderately or as well differentiated) and 122 (17.4%) were classified as high-grade (Gleason sum ≥ 7 or equivalent, *i.e*. coded as poorly differentiated or as undifferentiated).

Each case patient was matched to one control participant, with the exception of cases from Umeå in Sweden, an EPIC-associated cohort in which case patients were matched to two control participants, selected at random among appropriate risk sets consisting of all male cohort members alive and free of cancer (except nonmelanoma skin cancer) at the time of diagnosis of the index case. An incidence density sampling protocol for control selection was used, such that controls could include participants who became a case later in time, while each control participant could also be sampled more than once. Matching criteria included: recruitment center, age at enrolment (±6 months), time of day of blood collection (±1 hr) and time between blood draw and last consumption of food or drinks (<3, 3–6, >6 hr). While these matching criteria are not all necessary for analyses of genotype, they are applied to allow the same sets to be used for analyses of multiple markers of risk, such as nested case control analyses of circulating concentrations of hormonal and nutritional biomarkers.

### Genotyping

Genotyping of the C/T-13910 *lactase* variant (rs4988235) was conducted using the TaqMan assay (Applied Biosystems) at Imperial College, London. The internal quality of genotype data was assessed by 2% blinded samples in duplicate and an intralaboratory concordance rate of greater than 97.2% was observed. Empty wells (containing water only) were also included on each plate. Hardy–Weinberg Equilibrium (HWE) checks have been performed among the controls, stratified by country. No deviation in HWE was observed (*p* > 0.05). The SNP was successfully genotyped in 97.8% of samples. In total, genotype data for the current analysis were available for 630 cases: 79 cases in Denmark, 179 in Germany, 9 in Greece, 50 in Italy, 22 in the Netherlands, 69 in Spain, 84 in Sweden and 138 in the UK.

### Biomarker assays

Measurements of circulating concentrations of several nutritional and hormonal biomarkers made for previous nested case control studies of prostate cancer in EPIC were available for a subset of participants in the current study. These analytes were phytanic acid, 25-hydroxyvitamin D, insulin-like growth factor 1 (IGF1), insulin-like growth factor binding protein 3 (IGFBP3), androstenedione, androstanediol glucuronide, testosterone, sex hormone binding globulin (SHBG), calculated free testosterone, enterodiol and enterolactone and details of the laboratory assays have been described elsewhere.^22^–^26^

### Statistical analysis

The baseline characteristics of participants by *LCT* genotype were compared using analyses of variance and multinomial logistic regression models for continuous and categorical variables, respectively, with adjustment for center of recruitment.

To investigate dairy product intake by the *lactase* C/T polymorphism, analyses of variance were conducted for *LCT* genotypes, with adjustment for recruitment center, in relation to consumption of milk, yoghurt, cheese, butter, dairy based deserts (including ice-cream), total dairy products, dairy protein, total fat, fat from dairy products, calcium intake and circulating concentrations of nutritional and hormonal analytes. Nonconsumers of dairy milk are defined as persons consuming <5 g/day milk and milk beverages.

Conditional logistic regression models were applied to calculate the relative risks (odds ratios) for prostate cancer in relation to the *lactase* C/T genotype. Likelihood ratio chi-square tests were used to test the main effect of genotype on risk (comparing models with genotype terms with a model with no genotype terms), and tests for trend (trend tests of genetic effect) were obtained by scoring *LCT* genotypes as 0, 1 and 2, respectively, according to the number of variant T alleles, and with homozygotes for the C allele as the reference group. The effects of potential confounders, other than matching criteria, which are controlled for by design, were examined by including additional regression terms in the logistic regression models. Potential confounders were chosen *a priori* as factors that may be associated with the risk of prostate cancer developing or being detected and were smoking (never, past, present), body mass index (BMI, kg/m^2^; in fourths), physical activity (inactive, moderately inactive, moderately active combined with active) (16), alcohol intake (<8, 8–15, 16–39, ≥40 g/day), marital status (married/cohabiting or not married/cohabiting) and education level (primary or none, secondary, degree level). For each of these variables a small proportion of values were unknown; these values were included in the analyses as a separate category.

For tests of heterogeneity in associations of the *lactase* C/T polymorphism with risk of prostate cancer across subgroups and by case characteristics, we used likelihood ratio tests to compare models with a cross-product term between the genotype trend test variable and subgroup membership with models without the cross-product term. Participants were divided into subgroups according to prostate tumor stage (localized or advanced); histologic grade (low grade or high grade); time to diagnosis (less than 4 years after blood collection, 4 or more years after blood collection); age at blood collection (<60 or ≥60 years); and country of recruitment (8 countries). For tests of heterogeneity of risk by case characteristic (tumor stage, histological grade and time to diagnosis), the controls in each matched set were assigned the characteristics of their case.

To put the results of this study into the context of previous research, we conducted a meta-analysis of our results together with the results of previously published studies. Studies were identified by searching PubMed and the reference lists of relevant articles by using the search terms lactase and prostate cancer. Summary relative risks were estimated as the weighted average of the study-specific RRs for men with the TC or TT *lactase* genotypes compared with those with the CC genotype, with weights determined by the inverse of the variance of each relative risk.

Statistical analyses were performed with the Stata 10 statistical software package.^27^ All tests of statistical significance were two-sided, and *p* values below 0.05 were considered significant.

## Results

630 men diagnosed with prostate cancer from recruitment until the end of follow-up and 873 matched participants without prostate cancer were included in the analyses. Their median age at blood collection was 60 years (range: 43–76 years). Prostate cancer diagnosis followed blood collection by a median of 3.5 years (range: <1 to 9.5 years) and the median age at diagnosis was 64 years (range: 47–82 years).

**Figure 1:**
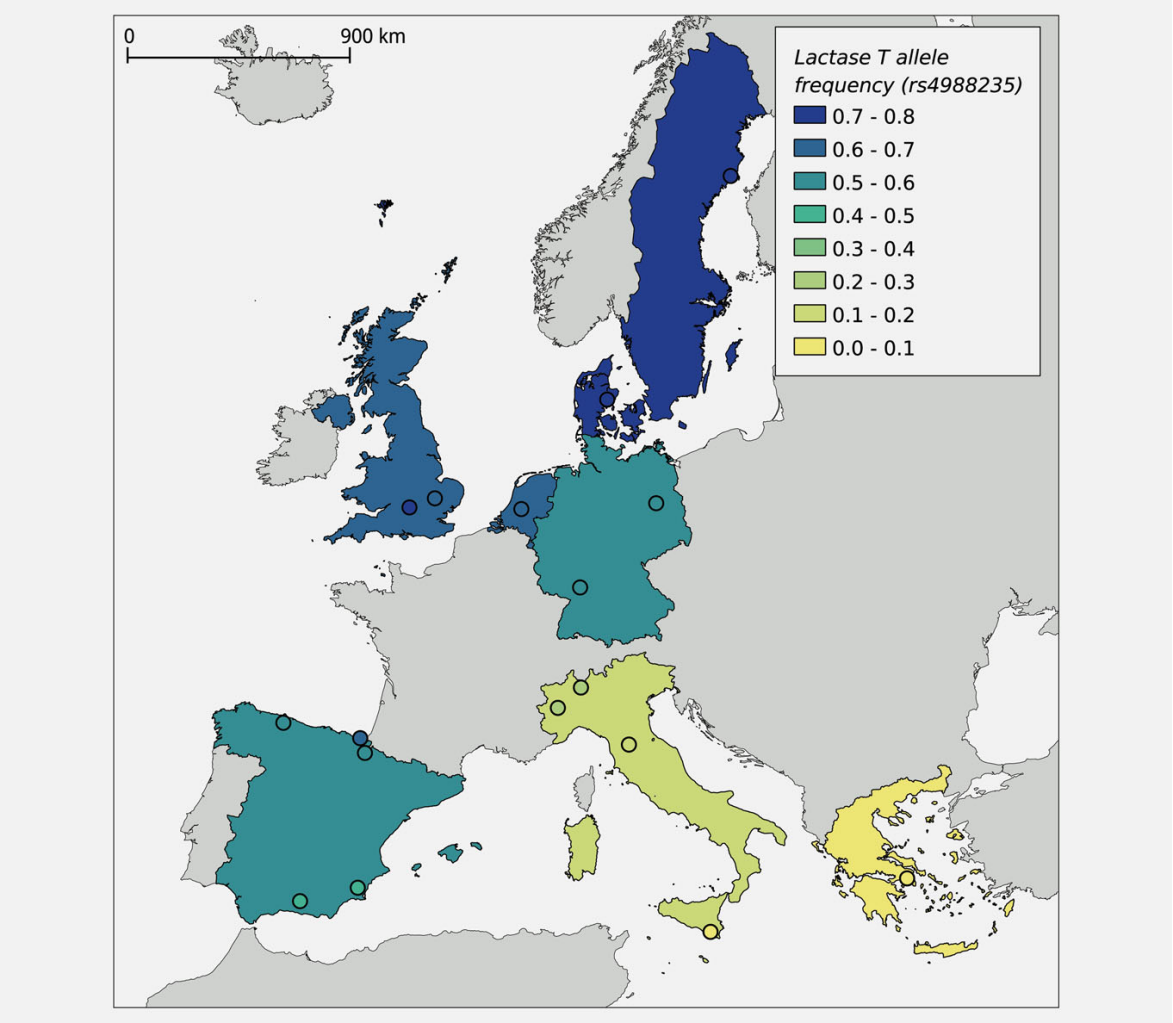
Frequency of the rs4988235 T allele by country in men from the European Prospective Investigation into Cancer and Nutrition. Color gradient indicates the average *lactase* T allele frequency in men from EPIC by country and by specific recruitment centers (indicated by filled circles).

Table 1 shows the distribution of the *lactase* genotypes in the eight countries participating in the study. Genotype frequencies followed the Hardy–Weinberg equilibrium (HWE) within each country (*p* > 0.05). Genotype frequencies varied markedly across Europe. In Greece none of participants and in Italy 5.6% of participants were homozygous for the T allele and 15% and 29% of participants, respectively, had at least one copy of the T allele. In contrast, in Sweden and Denmark, 55.6% and 63.3%, respectively, were homozygous for the T allele, and 95% and 94% of participants, respectively, had at least one copy of the T allele. Similarly, there was significant variation in allele frequencies between the countries, with frequencies of the T allele ranging from 7% and 17% in Greece and Italy, respectively, to 75% in Sweden and 79% in Denmark (Fig. 1). These genotype and allele frequencies were similar to published frequencies for corresponding regions of Europe.^10^,^13^–^15^

**Table 1:** Baseline characteristics by *lactase* genotype among all participants

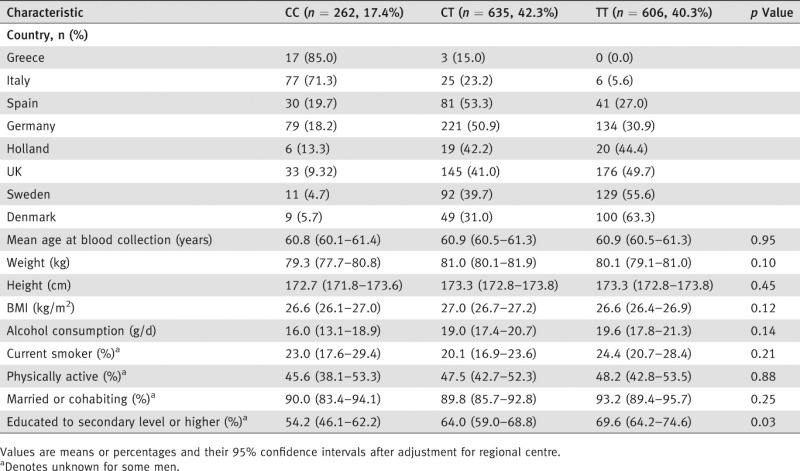

The baseline characteristics of participants by *lactase* genotype are shown in Table 1. After adjustment for recruitment center, the characteristics of participants did not vary significantly by genotype, with the exception of educational attainment; CC homozygotes were less likely to have been educated to secondary level or higher (54.2% educated to secondary level or higher) than were heterozygotes and TT homozygotes (64.0% and 69.6%, respectively).

The intake of dairy products and selected nutrients by *lactase* genotype after adjustment for recruitment center is shown in Table 2. Mean intake of milk, total dairy products and calcium increased significantly and the proportion of nonconsumers of milk decreased significantly with increasing number of copies of the *LCT* T allele. Adjusted mean intake of milk was 44.4 g/day among CC homozygotes, 69.8 g/day among heterozygotes and 82.3 g/day among TT homozygotes. There was no statistically significant heterogeneity in the relationship between milk intake and *LCT* genotype by country or recruitment center. Furthermore, even within the largest centers that had recruited participants from a small geographical area there was some evidence of a stepwise relationship; for example, in EPIC-Norfolk, intake of milk was 214.3 g/day among CC homozygotes, 297.8 g/day among heterozygotes and 309.1 g/day among TT homozygotes. Mean intake of protein from dairy products was also higher among *LCT* heterozygotes and TT homozygotes, respectively, although this association only reached borderline significance. No significant associations were observed between *LCT* genotype and intakes of yoghurt, cheese, butter, dairy desserts, dietary fat, or fat from dairy products. Mean intake of ice cream (a component of dairy desserts) was low in all *LCT* genotype groups, and while there was a statistically significant association between *LCT* genotype and ice cream intake (*p* value for difference = 0.02), with greater intake among heterozygotes, there was no significant trend in intake by genotype (*p* trend = 0.4).

**Table 2:** Intake of selected nutrients and dairy products by *lactase* genotype

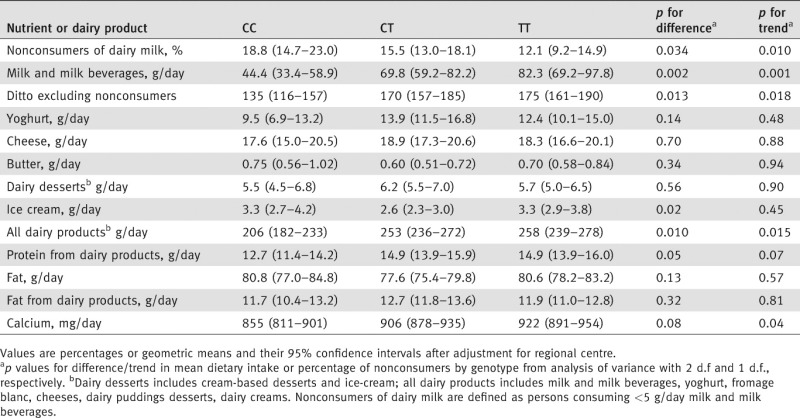

Table 3 shows the mean concentrations of selected blood analytes by *lactase* genotype. After adjustment for regional center, there was significant variation by genotype in the circulating concentration of phytanic acid and borderline significant variation in circulating concentrations of 25-hydroxyvitamin D and enterodiol, respectively, with higher levels of phytanic acid and 25-hydroxyvitamin D being observed among men with more copies of the *LCT* T allele and lower levels of enterodiol among carriers of the T allele. No significant association was observed between circulating concentrations of IGF1, IGFBP3, sex hormones, SHBG or enterolactone and *LCT* genotype.

**Table 3:** Geometric mean concentration of selected blood analytes by *lactase* genotype

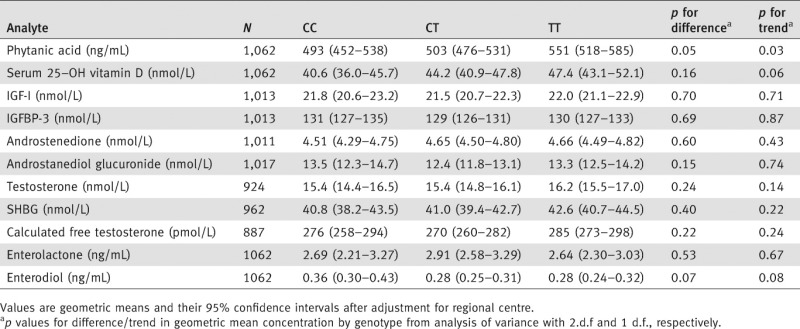

In participants with prostate cancer, the frequency of the *lactase* T allele was 40% and in control participants it was 40.5%. Table 4 shows relative risk of prostate cancer by *lactase* genotype. We observed no statistically significant association between risk for prostate cancer and *lactase* genotype, with or without adjustment for potential confounders in the recessive, codominant (additive) or dominant models. The adjusted odds ratios among CT heterozygotes and TT homozygotes compared with CC homozygotes were 1.03 (95% CI: 0.72–1.46) and 1.10 (0.76–1.59), respectively. There was no statistical evidence of heterogeneity in the trends in risk for prostate cancer by genotype according to the age of the men at blood collection, country of recruitment, prostate tumor stage, histological grade, or time to diagnosis (data not shown). The T allele frequencies were particularly low in Italy and Greece and therefore, in a sensitivity analysis, we also investigated the associations between the *LCT* variant and prostate cancer risk after excluding Greece and Italy; findings were not materially altered (data not shown).

**Table 4:** Relative risk^a^ (95% confidence interval) of prostate cancer by *lactase* (*LCT*) genotype in EPIC

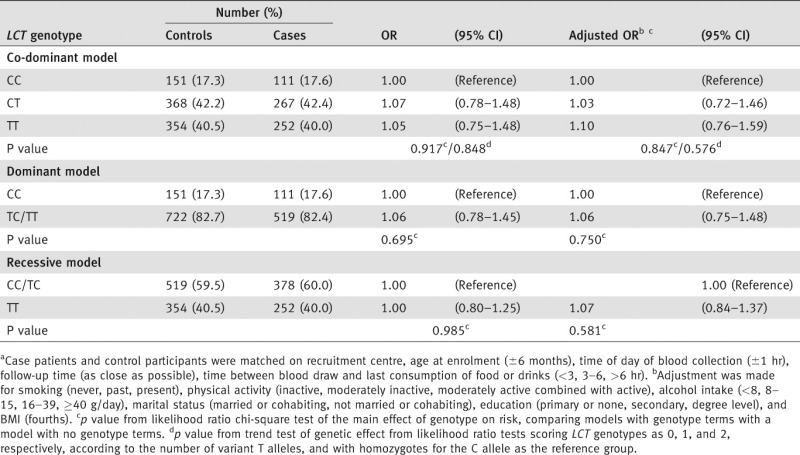

Results from the one previous study of the *LCT* variant and prostate cancer risk,^15^ together with the corresponding result from this study and a summary relative risk are shown in Table 5. In Swedish and Finnish populations, respectively, the *lactase* variant was not associated with a significantly increased risk of prostate cancer.^15^ Taking our results with these previous findings, the combined odds ratio for prostate cancer in men with at least one copy of the *lactase* T allele compared to men with no copies was 1.12 (95 % CI: 0.96–1.32).

**Table 5:** Relative risks (and 95% confidence intervals) from studies of the C/T-13910 *lactase* variant (rs4988235) in relation to prostate cancer risk, comparing carriers of the T allele (CT and TT genotypes) with CC homozygotes^a^

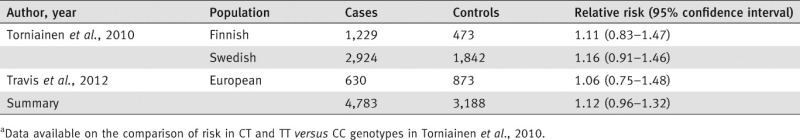

## Discussion

We investigated common genetic variation in the *lactase* gene in relation to intake of dairy products and risk for prostate cancer in a large, European study (EPIC). Our results provide evidence for a significant association of the C/T-13910 *lactase* variant with intake of milk but no evidence for a large association between the polymorphism and risk for prostate cancer.

The marked gradient in *LCT* allele frequency across Europe, both between and within countries, which we and others have observed^10^,^12^–^15^,^28^ coincides with substantial variation in diet and lifestyle practices between regions, including dairy product intake, and thus the overall relationship between *LCT* genotype and dairy product intake across Europe may be due to this concomitant cultural variation. Even in the absence of a true diet-genotype association, confounding due to this population stratification (*i.e*. systematic differences in allele frequency by region) might give rise to an apparent association between diet or biomarker concentrations and *lactase* genotype in EPIC overall. However, even after adjusting for the regional recruitment center we found that milk consumption, total dairy product intake and calcium intake were related to *LCT* genotype, with evidence for a dose–response relationship with increasing milk and calcium intake with a higher number of T alleles. Our observations of a slightly weaker trend in the intake of dairy protein by *LCT* genotype is consistent with the hypothesized mechanism of lactose intolerance given that dairy product intake predominantly comprises milk by weight, whereas dairy protein as a percentage of energy intake comprises both protein from lactose-rich milk and protein from the protein-dense but low lactose cheese.

Previously published epidemiological data on the *lactase* variant and milk intake are relatively limited and inconsistent. Our results are in accord with data from 1673 men in the Swedish CAPS study, which reported lower milk intake in CC homozygotes (221 g/day) compared to men carrying the T allele (348 g/day).^15^ However, they differ from the findings of a study on 643 Italian men and women from the EPIC-Italy cohort, a small number of whom may be included in the current analyses; while results from this Italian study showed a significantly higher intake of ice-cream among TT homozygotes, there was less evidence for an association between LCT genotype and milk intake.^13^ The results from two other studies, which had information on milk consumption (never vs. usually drink milk) but not on amount of milk consumed, were also inconsistent.^14^,^18^ In a study of 3,344 women from the United Kingdom, the likelihood of never drinking milk was not found to be associated with *lactase* genotype.^14^ In contrast, among 2396 men and women from Russia, Romania, Poland and the Czech Republic, a higher proportion of CC homozygous individuals reported never consuming milk than did carriers of the T allele.^18^ It remains possible that the overall association we observed between milk consumption and *LCT* genotype is due to residual confounding within center, particularly for large centers recruiting over wide geographical areas within which there may be variation in the frequency of the *lactase* C/T allele. However, our findings of no heterogeneity in the relationship between genotype and milk intake by recruitment center, and of associations among men from regional centers which recruited from geographically limited areas, such as EPIC-Norfolk, provide evidence in support of a true relationship between *LCT* genotype and milk consumption.

While the traditional view has been that lactase-persistence is an autosomal dominant trait, our results suggest a stepwise relationship, with a higher milk intake being observed with increasing number of T alleles. Our findings are consistent with those from a clinical study of 52 adults, in which lactase expression in intestinal biopsy samples was found to follow a trimodal distribution.^29^ The biopsy study found very low levels of lactase activity in individuals who were homozygous for the C allele.^29^ Our data also suggest that, as has been reported in other studies, individuals with the CC genotype can tolerate some milk consumption.

We also assessed whether the *lactase* variant was associated with circulating concentrations of a number of nutritional and hormonal biomarkers that were available for men in the current study, including phytanic acid, 25-hydroxyvitamin D, IGF1, enterodiol and enterolactone.^22^–^26^ Plasma concentrations of phytanic acid varied significantly by *LCT* genotype; higher concentrations were observed in TT homozygotes than in heterozygotes and CC homozygotes. These findings are consistent with the fact that phytanic acid is predominantly obtained from foods high in ruminant fat (*i.e*. meat and dairy products from cows, sheep and goats) and provide further indirect support for an association between *lactase* genotype and dairy food intake. We also observed borderline significant associations between *lactase* genotype and circulating concentrations of 25-hydroxyvitamin D and enterodiol; a higher concentration of 25-hydroxyvitamin D was observed with increasing number of copies of the *LCT* T allele and a lower concentration of enterodiol was observed among carriers of the T allele compared to CC homozygotes. Given the multiple statistical tests these may be chance findings and require confirmation in other large studies. In the one previous report on circulating vitamin D levels, no association was found with *lactase* genotype in two elderly Dutch populations (comprising 6,367 and 844 individuals, respectively).^30^ It is plausible, however, that there is an increased conversion of 25-hydroxy-vitamin-D into 1,25-hydroxy-vitamin D (the hormonal biologically active form of vitamin D) in individuals who have a lower calcium intake because they carry the *LCT* C allele. There might also be some degree of general malabsorption associated with the C allele, which means that there is lower absorption of dietary vitamin D into the body. An association between the *lactase* variant and enterodiol is also possible, given that differences in the digestion of lactose due to the *LCT* variant may alter the activity or composition of the gut microflora, which is responsible for the production of enterodiol from the plant lignan secoisolariciresinol.^31^ Intake of dairy products and calcium has been positively associated with circulating concentrations of insulin-like growth factors;^32^ however, we found no evidence for an association between *lactase* genotype and serum concentrations of IGF1 and IGFBP3.

Our findings of no significant association between *LCT* genotype and prostate cancer risk are consistent with those from the only previously published study of the *LCT* variant in relation to risk for prostate cancer, which reported no significant associations in nested case control studies among Finnish men (1,229 men with prostate cancer and 473 controls) and Swedish men (2,924 men with prostate cancer and 1,842 controls).^15^ Meta-analysis of our results together with these previous findings suggest that the lactase variant is not likely to be strongly related to prostate cancer risk, although it is not possible to exclude a small association (summary relative risk 1.12, 95% confidence interval 0.96–1.32). These data provide little support for a strong relationship between dairy product consumption and prostate cancer risk, consistent with the null findings in some but not all of the published studies on the association between dairy product consumption and prostate cancer risk.^2^–^7^,^33^–^38^ It is possible, however, that the relatively small average difference in milk intake between genotype groups (37 g/day between CC and TT homozygotes in EPIC overall, and 81 g/day in the UK where the difference was most marked) is of insufficient magnitude to have a detectable influence on risk for prostate cancer should such an association truly exist. Furthermore, it may be that other components of dairy foods are associated with prostate cancer. The *lactase* genotype is a marker of intake of lactose-rich dairy products (for example, milk, yoghurt, ice cream, low fat cream) rather than being a marker of dairy product intake *per se*, and is not correlated with dietary factors such as intake of cheese or butter.

Dairy intake and the *lactase* gene-variant in relation to prostate cancer has been one of the specific hypotheses discussed with respect to the application of Mendelian Randomization (MR) to assess relationships between environmental exposures and risk for disease.^14^ The current analyses, however, demonstrate some of the challenges of applying Mendelian Randomization to explore the relationship between dietary factors and cancer risk.^39^ Because the expected association between dairy products and prostate cancer is relatively small in magnitude and the difference in total dairy intake by *lactase* genotype is modest, even in this large prospective study and with a relatively common allele of interest (the frequency of the T allele was 0.61 overall), there is relatively limited power to detect an association between the *lactase* genotype and the disease. Based on previous findings from a prospective cohort analysis in EPIC^1^ for example, in which a 35 g per day increment in dairy protein intake was associated with an approximately 32% increase in risk for prostate cancer, the 2.2 g per day difference in dairy protein intake between CC and TT homozygotes would be estimated to result in only a 2% elevated risk among TT homozygotes compared with CC homozygotes. With the *LCT* genotype frequencies observed in EPIC, a study of approximately 30,000 cases and 30,000 controls would be needed to detect a relative risk for prostate cancer of this magnitude with 80% power.

The results from this large prospective study in European men suggest that variation in the *lactase* gene is associated with intake of milk but provide no evidence for a large association with risk for prostate cancer. The data presented illustrate the limitations of applying Mendelian Randomization as a research strategy to explore the relationship between dairy product consumption and cancer risk and the need for very large prospective studies with both genetic and dietary data in such studies of genetic proxies of nutritional exposures.
